# Immunogenicity of pulsatile-release PLGA microspheres for single-injection vaccination

**DOI:** 10.1016/j.vaccine.2017.05.094

**Published:** 2018-05-24

**Authors:** Rohiverth Guarecuco, Jennifer Lu, Kevin J. McHugh, James J. Norman, Lavanya S. Thapa, Emily Lydon, Robert Langer, Ana Jaklenec

**Affiliations:** David H. Koch Institute for Integrative Cancer Research, Massachusetts Institute of Technology, 500 Main Street, Cambridge, MA 02142, USA

**Keywords:** Vaccine delivery, Controlled release, Single-injection vaccines, Immune response, Microparticles, Biodegradation, Poly(lactic-co-glycolic acid)

## Abstract

The World Health Organization's Expanded Programme on Immunization has led to a dramatic rise in worldwide vaccination rates over the past 40 years, yet 19.4 million infants remain underimmunized each year. Many of these infants have received at least one vaccine dose but may remain unprotected because they did not receive subsequent booster doses due to logistical challenges. This study aimed to develop injectable controlled release microparticles with kinetics that mimic common vaccine dosing regimens consisting of large antigen doses administered periodically over the course of months in order to eliminate the need for boosters. Sixteen poly(lactic-co-glycolic acid) (PLGA) microsphere formulations containing bovine serum albumin (BSA) as a model vaccine antigen were screened in vitro to determine their respective release kinetics. Three formulations that exhibited desirable pulsatile release profiles were then selected for studying immunogenicity in mice. Two low-dose microsphere formulations induced peak anti-BSA IgG antibody titers of 13.9 ± 1.3 and 13.7 ± 2.2 log_2_ compared to 15.5 ± 1.5 log_2_ for a series of three bolus injections delivered at 0, 4, and 8 weeks with an equivalent cumulative dose. Similarly, high-dose formulations induced peak antibody titers that were 16.1 ± 2.1 log_2_ compared to 17.7 ± 2.2 log_2_ for controls. All three microparticle formulations studied in vivo induced peak antibody titers that were statistically similar to bolus controls. These results suggest that pulsatile antigen release from polymeric microparticles is a promising approach for single-injection vaccination, which could potentially reduce the logistical burden associated with immunization in the developing world.

## 1 Introduction

Despite the immense increase in vaccine coverage worldwide over the past four decades, vaccine-preventable infectious diseases still claim the lives of approximately 1.5 million children each year [1]. However, these deaths are not due to inadequate vaccine function, but rather inadequate distribution and administration of vaccines – especially in some areas of the developing world. Although nearly 86% of infants are fully immunized against diphtheria, tetanus, and pertussis, 19.4 million infants remain underimmunized against these pathogens [2]. Of these infants, 6.6 million have received at least one dose of the vaccine, but remain at-risk because they did not receive a full series of doses (DTaP3) due to limited healthcare access or other socioeconomic factors [3], [4], [5]. Unfortunately, a single bolus administration is not typically adequate to ensure robust and durable immunity [6].

Microparticle-based controlled release of vaccines may present an option for achieving immunity after only one administration [7]. These devices, which release antigen over time, could eliminate need for booster injections, thereby reducing the logistical barrier by two-thirds and completely eliminating dropout for many vaccines [8]. Over the past 35 years, researchers have attempted to create polymeric systems capable of extended antigen release to provide immunity after only one injection [9]. Poly(lactic-co-glycolic acid) (PLGA) microparticles have been widely used in these systems owing to their precedence in existing biomedical products and tunable release kinetics [10], [11]. These microspheres can be delivered in a single injection and release their contents over days, weeks, or months depending on their properties. Further, depending on their composition and fabrication parameters, PLGA microspheres can be designed to obtain near zero-order, first-order, or pulsatile release kinetics [12], [13], [14], [15], [16], [17], [18]. Although there is some evidence that alternative antigen presentation kinetics result in strong immune responses [19], [20], pulsatile antigen release that best mimics bolus dosing regimens known to be safe and effective may be desirable [21].

Several groups have reported on pulsatile release from PLGA microspheres in vitro [16], [17], [18], but equivalent in vivo studies have only begun recently [22]. Herein, we describe the development, in vitro release kinetics, and in vivo immunogenicity of PLGA microsphere formulations that release bovine serum albumin (BSA) in a series of pulses after administration. Pulsatile microsphere development focused on utilizing the inherent bulk eroding properties of PLGA, which yield tri-phasic release kinetics [21], [23]. The initial burst can be attributed to the release of antigen from the microparticle surface, the second to antigen diffusion through porous microparticles, and the third to antigen release during structural degradation of microparticles. We hypothesized that by changing polymer composition (e.g. lactic-to-glycolic acid ratio, end group), polymer molecular weight, and antigen loading, we could adjust these bursts to occur at desired intervals. Serum antibody titers from animals treated with BSA-loaded microspheres were compared to those from animals treated with a series of bolus BSA injections representative of a common immunization schedule.

## 2 Materials and methods

### 2.1 Materials

Poly(D,L-lactic-co-glycolic acid) (PLGA Resomer® RG 502 H, RG 503 H, RG 504 H, and RG 752 H) and BSA were purchased from Sigma-Aldrich (St. Louis, MO). Poly(vinyl alcohol) (PVA, Mw = 25,000) was purchased from Polysciences, Inc. (Warrington, PA). Dichloromethane (DCM) and 2,2,2-trifluoroethanol (TFE) used in this study were reagent grade.

### 2.2 Microsphere fabrication

Sixteen formulations of PLGA microspheres containing BSA (Table 1) were fabricated using a spontaneous single-emulsion/solvent evaporation method previously reported [24], [25]. Briefly, 200 mg of PLGA were dissolved in 10 mL of 4:1 DCM:TFE and mixed with 300 µL of BSA in water. Mixing formed a clear, single-phase solution that was subsequently added to 200 mL of 5% (w/v) PVA in water. The emulsion formed spontaneously and was stirred for 3 h at room temperature. Particles were then centrifuged, washed five times with water, and lyophilized. When prepared for in vivo use, PLGA and BSA solutions were filtered through 0.2 µm polytetrafluoroethylene filters (Whatman, Little Chalfont, England) prior to forming the emulsion and mixed in a sterile laminar flow hood.

**Table 1:** Microsphere formulations and size characterization.

Formulation	BSA (% w/w)	PLGA M_w_ (kDa)	PLGA ratio	Particle size (µm)	90% Threshold diameter^a^ (µm)
A	5	7–17	50:50	10.5 ± 6.8	18.5
B	3	7–17	50:50	10.6 ± 6.4	18.1
C	0.5	7–17	50:50	10.3 ± 6.2	22.1
D	0	7–17	50:50	10.5 ± 5.9	17.4
E	5	24–38	50:50	8.6 ± 6.7	21.4
F	3	24–38	50:50	11.4 ± 8.3	21.4
G	0.5	24–38	50:50	12.1 ± 8.2	23.1
H	0	24–38	50:50	11.4 ± 7.2	20.2
I	5	38–54	50:50	14.1 ± 9.4	25.4
J	3	38–54	50:50	12.0 ± 7.1	20.3
K	0.5	38–54	50:50	11.9 ± 6.8	20.6
L	0	38–54	50:50	11.9 ± 6.4	19.7
M	5	4–15	75:25	11.3 ± 7.0	19.7
N	3	4–15	75:25	12.2 ± 7.4	21.2
O	0.5	4–15	75:25	12.4 ± 7.5	21.1
P	0	4–15	75:25	11.7 ± 6.5	19.9

a 90% of particles in the formulation are smaller than this threshold.

### 2.3 Microsphere characterization

Microsphere size distribution was determined using a Multisizer 3 Coulter Counter (Beckman Coulter, Brea, CA). Histograms were created using a bin size of 0.39 µm and smoothed using central moving average with a window size of ±5 bins. Scanning electron microscope (SEM) images were collected using a JSM-5600LV SEM (JEOL, Tokyo, Japan) at an acceleration voltage of 5 kV. Prior to imaging, samples were coated with Au/Pd using a Hummer 6.2 Sputtering System (Anatech, Battle Creek, MI) to prevent surface charging.

### 2.4 In vitro BSA release

Ten milligrams of microspheres were dispersed into 1 mL phosphate-buffered saline (PBS) in capped tubes and incubated on a rotating platform at 8 RPM and 37 °C. At each time point (day one, then weekly for 1–13 weeks), samples were centrifuged at 1500 RCF for 5 min, after which the supernatant was collected. Samples were then resuspended in fresh PBS and returned to the incubator for sampling at subsequent time points. BSA release from microspheres was quantified using a BCA assay kit (Thermo Scientific Pierce, Rockford, IL) and normalized to the total amount released by the end of the study. Samples were run in triplicate and data reported as mean ± standard deviation.

### 2.5 In vivo administration of BSA microspheres

All animal work was approved by MIT’s Committee on Animal Care. Briefly, female BALB/c mice 6–8 weeks of age received injections of (1) BSA-loaded microspheres, (2) empty microspheres, (3) bolus BSA, or (4) saline. While mice in the first two groups received only one injection, those receiving a bolus BSA or saline only were injected again at 4 and 8 weeks to match the amount and timing of BSA release from PLGA microspheres in vitro. Twenty mg of BSA-containing PLGA microparticles were suspended in 400 µL of saline and half of the solution was injected subcutaneously into each hind limb. At this particle mass, mice in the low dose (0.5%) groups received 64 µg (Formulation C) or 71 µg (Formulation G) of total encapsulated BSA, while mice in the high dose (5.0%) group (Formulation E) received 431 µg. Control mice received similar subcutaneous injections of soluble BSA such that the total cumulative dose approximated the amount of BSA released in vitro by microparticle formulations for the low and high loading groups. Table 2 contains the exact dosing regimen for each group. At week 0, 1, 2, 4, 6, 8, and 10, 100 µL of blood was sampled sub-mandibularly and, after clotting, was centrifuged at 2000 RCF for 10 min at 4 °C to separate the serum.

**Table 2:** Dosing regimen for in vivo antigen administration.

	Amount of BSA (µg)			
Group	First dose	Second dose	Third dose	Total
Formulation C (0.5% BSA, 7–17 kDa PLGA)^a^	22	20	22	64
Formulation G (0.5% BSA, 24–38 kDa PLGA)^a^	23	14	34	71
Low Dose Bolus BSA	22	22	22	66
Formulation E (5% BSA, 24–38 kDa PLGA)^a^	298	68	65	431
High Dose Bolus BSA	298	68	65	431
Empty PLGA microspheres, 7–17 kDa	0	0	0	0
Empty PLGA microspheres, 24–38 kDa	0	0	0	0
Saline	0	0	0	0

All PLGA used in vivo was 50:50.

a Indicates theoretical dose based on in vitro results.

### 2.6 Immunogenicity of BSA released from PLGA microspheres

Serum antibody titers against BSA were determined using an endpoint enzyme-linked immunosorbent assay (ELISA). ELISA plates ((96-well Maxisorp, Thermo Fisher Scientific, Waltham, MA) were coated overnight at 4 °C with 100 μL of a 100 μg/mL solution of BSA in 0.1 M sodium bicarbonate buffer, pH 9.5. Plates were then washed three times in PBS containing 0.05% Tween 20 (PBST) and incubated in 5% non-fat milk in PBST for 2 h at 37 °C as a blocking agent. Following another series of three washes with PBST, mouse serum samples were added in fourfold serial dilutions and incubated for 2 h at 37 °C. The extent of serum dilution increased as the study progressed and titers rose. Plates were then washed five times with PBST and incubated at 37 °C with horseradish peroxidase-conjugated goat anti-mouse secondary antibody (Southern Biotechnology Associates, Birmingham, Alabama) diluted 1:1000 in blocking buffer for 2 h. Plates were washed an additional five times with PBST and developed using 100 μL of *p*-nitrophenyl phosphate solution prepared from tablets dissolved in 1x diethanolamine buffer from an alkaline phosphate substrate kit (Bio-Rad, Hercules, CA). After 10 min, the reaction was stopped by adding 100 μL of 0.4 M sodium hydroxide to each well, and absorbance values were read at 405 nm using a Tecan Infinite M200 Pro microplate reader (Männedorf, Switzerland). Titers were reported as the reciprocal of the highest serum dilution that yielded an absorbance greater than 2-fold above background values.

### 2.7 Statistical analysis

All data are reported as mean ± standard deviation. In vitro studies were performed with n = 3 while in vivo studies were performed with n = 10, except for Formulation C at week 10, in which n = 9 due to insufficient blood volume from one animal. Antibody titers within each experimental treatment group were compared using Student’s paired two-tailed *t*-test. Antibody titers and time-to-peak comparisons between groups were analyzed using Student’s unpaired two-tailed *t*-test. The Holm-Bonferroni method was used in comparing peak antibody titers to counteract the effect of multiple comparisons.

## 3 Results

### 3.1 Characterization of BSA-containing PLGA microspheres

**Fig. 1:**
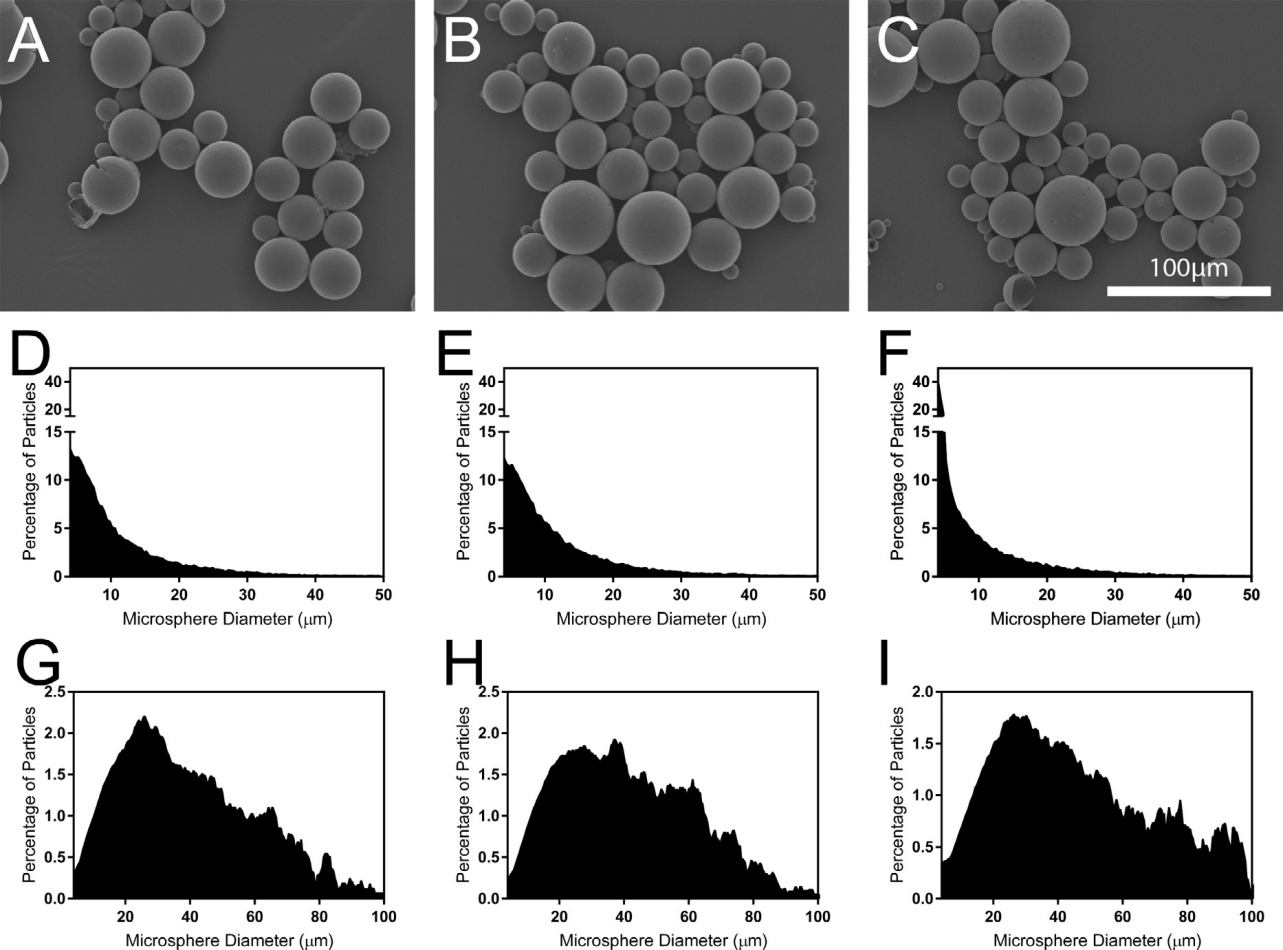
Characterization of BSA-containing PLGA microspheres. Scanning electron microscopy images of particles formed with (A) Formulation C, (B) Formulation G, and (C) Formulation E, demonstrating spherical and smooth morphology. Small microspheres may be underrepresented in these images due to their tendancy to accumulate beneath the curvature of larger particles during sample preparation. Histograms depiciting the size distribution of microspheres prepared with (D) Formulation C, (E) Formulation G, and (F) Formulation E as well as the volume distribution of (G) Formulation C, (H) Formulation G, and (I) Formulation E microspheres.

All sixteen formulations of PLGA and model vaccine antigen (BSA) produced spherical microparticles with broad distributions of particle sizes (Table 1). The size and shape of Formulations C, G, and E, which were used for in vivo studies, were representative of all formulations (Fig.1A–C), suggesting that particle size did not play a major role in immunogenicity. Formulation C produced microspheres that were 10.3 ± 6.2 µm in diameter; however, particles smaller than 10.3 µm contained just 4.2% of the antigen load due to the cubic growth of volume with diameter, assuming homogeneous distribution of BSA in PLGA [26]. Larger particles contained a majority of the antigen, with 90% of the volume contained in particles larger than 22.1 µm for Formulation C. Formulations G and E demonstrated similar characteristics, with particle diameters of 12.1 ± 8.2 and 8.6 ± 6.7 µm, respectively, yet with 90% of particle volume contained in particles larger than 23.1 and 21.4 µm, respectively. Of the formulations studied in vivo, Formulation E had the highest proportion of very small particles with 40.1% falling within the smallest bin size (4 µm), Histograms of microsphere diameter and volume distribution can be seen in Fig.1D–I. Particles at the large end of the distribution also contributed substantially to surface area effects as 50% of the total particle surface area for Formulations C, G, and E were contributed by particles larger than 23.7, 29.5, and 27.6 µm in diameter, respectively.

### 3.2 In vitro release of BSA from PLGA microspheres

**Fig. 2:**
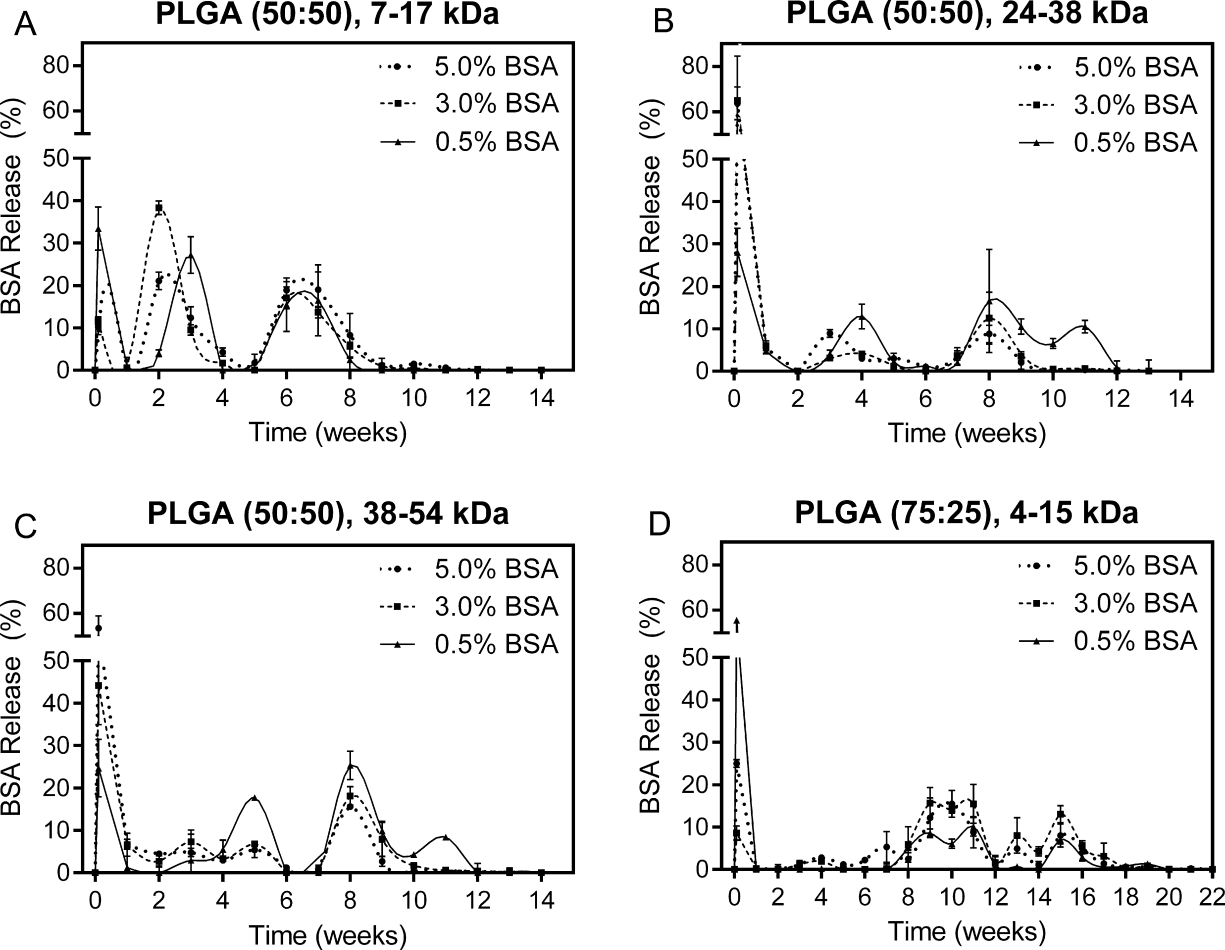
Weekly BSA release from microspheres formulated with (A) 7–17 kDa, 50:50 PLGA, (B) 24–38 kDa, 50:50 PLGA, (C) 38–54 kDa, 50:50 PLGA, and (D) 4–15 kDa, 75:25 PLGA containing 0.5, 3, or 5% BSA by weight. All data normalized to the total amount of protein recovered throughout the experiment. Error bars represent standard deviation.

In vitro release kinetics from PLGA microsphere formulations were determined using a bicinchoninic acid (BCA) assay and expressed as a percentage of the total BSA released during the duration of the experiment. BSA release and particle degradation occurred more quickly in PLGA microsphere formulations with copolymer ratios of 50:50 lactic-to-glycolic acid compared to a 75:25 copolymer ratio. All microsphere formulations produced using PLGA with a 50:50 ratio degraded within 14 weeks, whereas 75:25 PLGA degraded in 22 weeks. The timing of BSA release appeared to be more dependent on polymer type than BSA loading, though loading had a major effect on the size of the bursts in most cases. Low molecular weight (7–17 kDa) PLGA microparticles released BSA in three distinct bursts over the course of 8 weeks and completely degraded by week 14, as seen in Fig.2A. Medium molecular weight (24–38 kDa) PLGA microparticles also degraded by week 14, but displayed three bursts spread over a longer period of time (9–12 weeks) depending on BSA loading (Fig.2B). Microparticles composed of the highest molecular weight (38–54 kDa) PLGA tested released BSA over 9–12 weeks with prominent bursts at day 1 and week 8; however formulations with 3% and 5% BSA loading also exhibited a continuous release kinetics between these bursts (Fig.2C). Microspheres made from low molecular weight (4–15 kDa) PLGA with a higher lactic acid content (75:25) degraded over a much longer period of time (22 weeks) despite the molecular weight and demonstrated gradual, semi-continuous release after an initial burst (Fig.2D).

Out of the sixteen in vitro formulations, Formulations C, G, and E were chosen for the subsequent in vivo study based on their in vitro release kinetics. Formulation C microspheres were fabricated using low molecular weight PLGA (7–17 kDa) loaded with 0.5% BSA, Formulation G with slightly higher molecular weight PLGA (24–38 kDa) and 0.5% BSA, and Formulation E with the same molecular weight PLGA as Formulation G, but with higher (5%) BSA loading. BSA release from Formulation C was characterized by three distinct peaks at day 1, when 33.4 ± 5.1% of total BSA was released, at week 4, when 27.2 ± 4.3% was released, and across the week 6 and week 7 time points, during which 32.1 ± 5.2% was released (Fig.2A). Minimal BSA release was observed at weeks 1, 2, 4, and 5, or after week 7, and microspheres were completely degraded by week 10 as evidenced by complete dissolution of the particles.

Formulation G microspheres were characterized by BSA release in four apparent bursts spread out over a longer timeframe (Fig.2B). In addition, total microsphere degradation was observed after 14 weeks rather than 10 weeks for Formulation C. The first BSA burst from Formulation G was observed at the one-day time point, releasing 28.0 ± 5.7% of total BSA. This was followed by a second burst of 12.9 ± 2.9% at week 4 and two overlapping bursts at weeks 8 through 11, during which a cumulative 43.8 ± 2.0% of BSA was released. Minimal BSA was observed in the release media at any other time points through complete degradation of the particles.

Formulation E microspheres released 63.7 ± 7.3% of its BSA at day 1, which was the largest initial burst in terms of both total quantity and percentage for any of the sixteen formulations (Fig.2B). The only time points with substantial BSA release following this initial burst were weeks 3 and 8 when 8.9 ± 0.9% and 8.8 ± 2.1% of the total load was released, respectively. The microspheres produced by this formulation degraded completely by 14 weeks similarly to Formulation G, which used PLGA at the same molecular weight (24–38 kDa).

### 3.3 Immunogenicity of BSA-containing PLGA microspheres

**Fig. 3:**
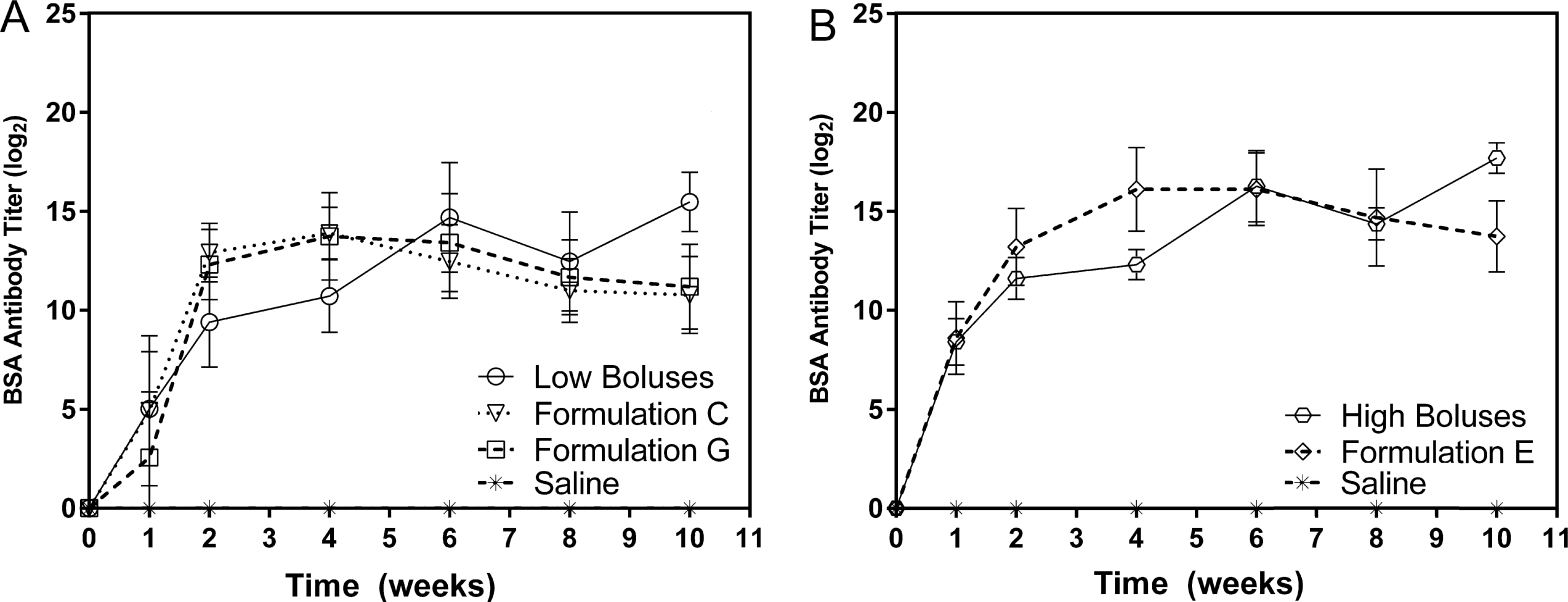
IgG antibody titers against the model antigen (BSA) from mouse sera plotted as geometric mean over time on a log_2_ scale. (A) Low-dose Formulations C & G compared to a series of three dose-matched bolus BSA injections and (B) high-dose Formulation E compared to its dose-matched bolus control. Bolus BSA was injected at 0, 4, and 8 weeks in the control groups.

The humoral immune response to each microsphere formulation was compared with a positive control consisting of three bolus injections approximating the quantity and timing of BSA released from particles in vitro. Within experimental groups, a statistically significant increase in titer between consecutive weeks was used as a surrogate indication of release. Formulation C induced a significant increase in antibody titers compared to the previous time point at weeks 1, 2, and 4 (p < 0.05, p < 0.001, and p < 0.001 respectively), then decreased significantly at weeks 6 and 8 (p < 0.01 and p < 0.05 respectively), before stabilizing at week 10 (Fig. 3). Similarly, mice receiving Formulation G showed a significant increase in titer at weeks 1, 2, and 4 (p < 0.05, p < 0.001, and p < 0.01 respectively), remained steady at week 6, and then fell significantly by week 8 (p < 0.05) before stabilizing again at week 10. Formulation E induced a similar response as antibody titers increased significantly at weeks 1, 2, and 4 (p < 0.001 for all), leveled off at week 6, and then decreased through the end of the study (p < 0.05). Overall, the immune response to all three microparticle formulations demonstrated a similar progression over time as titers rose over the first 4 weeks then slowly decreased through week 10 (Fig. 3). However, the magnitude of antibody titers appeared highly dependent on BSA loading. Based on in vitro results, Formulation E released approximately 13 times more BSA than Formulation C at the earliest time point (1 day) due to a large initial burst and induced antibody titers that were 13-fold higher as well. This trend was also observed at the end of the in vivo study where Formulation E released 7 times the amount of antigen compared to Formulation C resulting in an 8-fold higher antibody titer.

Although antibody titers are frequently compared to time-matched controls, comparing peak antibody titers may be a more useful indicator of immunogenicity since they enable comparison between groups whose antigen release/administration occurs at different times [27], [28]. Although the bolus injection schedule was chosen to match the timing of microsphere bursts based on in vitro results, accelerated in vivo degradation likely accelerated antigen release. Antibody titers in both groups receiving bolus BSA injections peaked at 8 ± 2 weeks. This was significantly later (p < 0.001) than observed in any of the groups receiving microspheres, which peaked at 4 ± 0, 4 ± 1, and 5 ± 1 weeks for Formulations C, G, and E, respectively. As a result, by the end of the experiment antibody titers in the microsphere groups had been falling for weeks (as would be expected in the absence of antigen [29], [30]), whereas a majority of animals in the bolus groups reached their highest antibody titer at 10 weeks following the third injection.

**Fig. 4:**
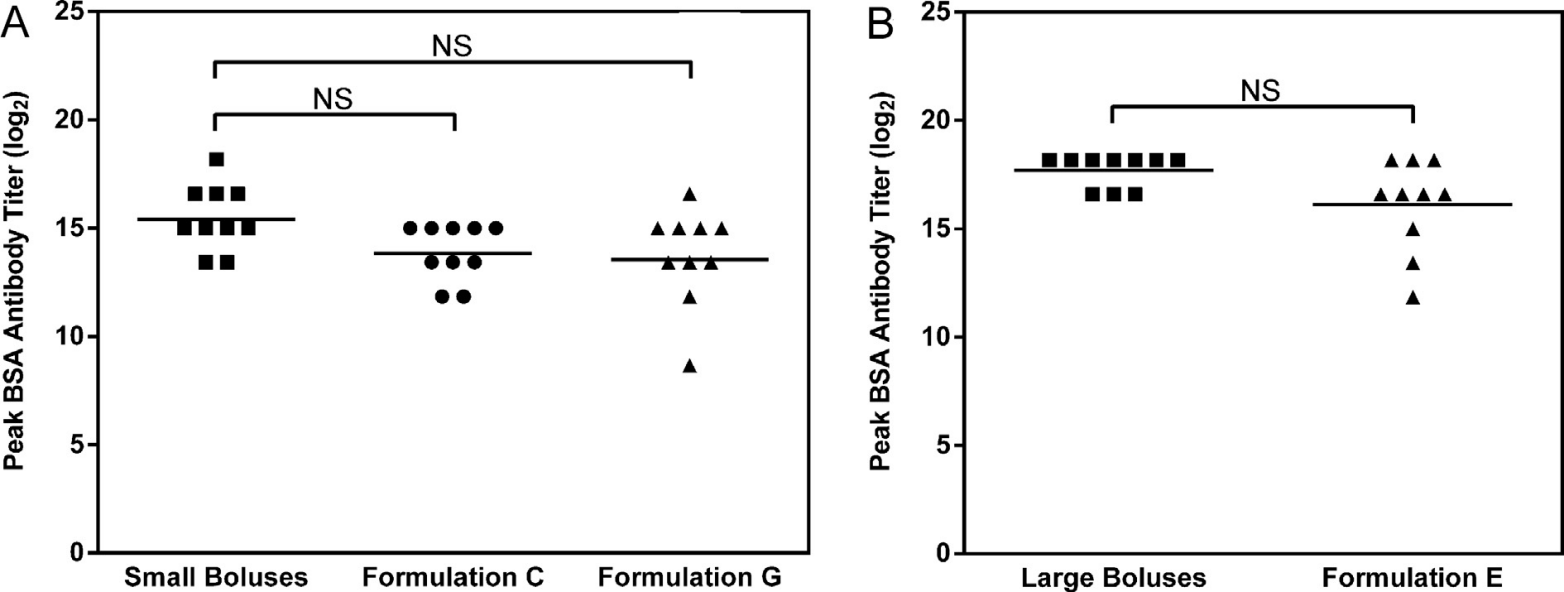
Peak titers induced by immunization at (A) low, and (B) high doses of model antigen (BSA). Peak titers for all microparticle formulations occurred at week 4 while both bolus injection groups peaked at week 10. NS represents not significant when using a Student’s *t*-test with Holm-Bonferroni correction for multiple comparisons at a significance level of 0.05. Adjusted p values for Formulations C, G, and E are 0.065, 0.054, and 0.078 respectively.

Antibody titers for groups treated with Formulations C, G, and E peaked at 13.9 ± 1.3, 13.7 ± 2.2, and 16.1 ± 2.1 on a log_2_ scale, respectively, 4 weeks after microsphere administration, whereas groups receiving the small and large dose-matched boluses peaked after 10 weeks at 15.5 ± 1.5 and 17.7 ± 0.8 log_2_ titer, respectively (Fig. 4). Formulations C and G (Fig.4A) induced peak antibody titers that were not statistically different (p = 0.065 and p = 0.054 respectively) from the dose-matched bolus control consisting of three 22 µg BSA injections, using the Holm-Bonferroni correction method that has been recommended for multi-group titer comparisons [31]. Formulation E (Fig.4B) also induced peak titers that were not statistically different (p = 0.078) from the dose-matched bolus control consisting of three bolus injections (Table 2).

## 4 Discussion

Several conclusions regarding antigen release rate and profile could be made based on the trends observed across the sixteen microsphere formulations studied in vitro. As expected, microsphere formulations fabricated using PLGA with 75:25 lactic-to-glycolic acid ratio degraded more slowly in vitro than 50:50 PLGA at a similar molecular weight [32]. In addition, the copolymer ratio of lactic-to-glycolic acid was observed to have a greater effect on degradation and antigen release kinetics than polymer molecular weight. From these initial formulations, Formulation C was selected for subsequent in vivo study due to its three evenly spaced, nearly equivalent bursts that approximate many current day vaccination regimens. Formulation E was chosen to investigate the effect on immunogenicity of a large primary antigen dose followed by two smaller doses. Lastly, Formulation G, with the same polymer as Formulation E and the same BSA content as Formulation C, was chosen to isolate the effects of polymer molecular weight and antigen loading on immunogenicity.

Each microparticle formulation contained microspheres of approximately equal size, and therefore surface area, which have been shown to affect immunogenicity elsewhere [33]. As a result, this study was largely able to isolate the effects of molecular weight (Formulation C vs. G) and antigen loading (Formulation G vs. E) on release kinetics and corresponding antibody titers. Despite the relatively high proportion of small (<10 µm) particles within all three formulations (Fig.1D–F), release kinetics were likely dominated by the sub-population of larger (>20 µm) microspheres since they contained a vast majority of polymer and presumably antigen as well (Fig.1G–I). However, the more numerous smaller particles may still play an important role in this system as microspheres <10 µm have been shown to enhance antigen immunogenicity via macrophage uptake and routing to the lymph nodes where B cells reside [34], [35].

Microsphere degradation and antigen release in vitro was similar to what others have previously reported for PLGA [18]. Particles formulated with lower molecular weight PLGA (7–17 kDa) degraded over the course of 10 weeks while higher molecular weight (24–38 kDa) degraded in 14 weeks. After the initial burst release of BSA from the particle surface, release continued as a consequence of polymer degradation and diffusion out of the hydrated polymer [13], [16]. Formulation E, which contained 10-fold more BSA than the other two formulations, released a majority of its antigen load in the first of the three bursts. In contrast, the amount of BSA released in the initial burst of Formulations C and G was approximately equal to the second and third bursts. This difference in initial burst from Formulation E is likely due to the greater amount of BSA at the particle surface and interconnectivity of protein cavities within the particle, which allow protein to diffuse out of the particle even before any meaningful polymer degradation has occurred [21].

Ultimately, the aim of this study was to mimic the antigen presentation timing of current multi-injection approaches using a single injection of controlled-release microparticles. Most current vaccines are administered in multiple, equal doses spread greater than one month apart and typically at least two months apart in non-emergency scenarios [36]. Formulation C released approximately one-third of its antigen at 0, 3, and 6 weeks in vitro, which is similar to current multi-injection vaccination regimens in terms of equivalent dose but insufficiently spaced to match many current vaccination schedules. Alternatively, Formulation G released antigen at 0, 4, and 8 weeks, in line with the minimum acceptable spacing for vaccination, but in unequal bursts [36]. However, several groups have suggested that a strategically varying antigen doses could actually improve immune memory [30], [37].

Results from in vivo evaluation of these formulations show that circulating antibody titers from mice treated with BSA-loaded PLGA microspheres reached a maximum after 4 weeks for all three formulations (Fig. 3), suggesting that nearly all BSA had been released by that time. This several-fold increase in apparent in vivo release rate has been previously reported and attributed to accelerated polymer degradation in vivo [17], [38]. The complete in vivo degradation of microspheres likely followed the same trend, meaning that degradation occurred in approximately 4–6 weeks rather than 10–14 weeks. This degree of acceleration in vivo is similar to other studies that have estimated microsphere degradation to be 1.7–2.6 times more rapid in vivo than in vitro [39] because of prolonged autocatalytic action of acid [17] as well as plasticization of PLGA by lipids and other biological molecules [40]. Antibody titers for the control groups receiving injections at 0, 4, and 8 weeks spiked following each bolus injection with progressively increasing maximum values, as expected. Despite potentially accelerated release in vivo, which could diminish the immune response, antibody titers induced by PLGA microparticle formulations were statistically similar to the three bolus dose-matched controls. This could be due, in part, to the inherent adjuvancy of the microparticles themselves. Several groups have shown that PLGA particles can serve as adjuvants via mechanisms that may involve a combination of immune cell recruitment at the injection site, macrophage routing to the lymphatic system, and prolonged persistence in the lymph node [41], [42], [43], [44].

Taken together, these results suggest that PLGA microparticles may be well-suited for the controlled release of vaccines and other protein therapies. Even with relatively low loading, the particle masses and injection volume administered here are in line with existing therapies. For example, Tripedia®, a DTaP vaccine, has a cumulative dose of approximately 47 µg [45], which would require about 14 mg of our 0.5% loaded particles. This is far less than the mass of PLGA microparticles currently administered in clinical formulations, which can exceed 1 g [46]. Likewise, the 400 µl injection volume used here is less than in the intramuscularly-administered Tripedia® (500 µl) as well as many current therapies injected subcutaneously, which can be as much as 2 ml [47]. While single-injection vaccines could potentially cost more than traditional vaccines to aseptically produce, this rise in cost may be more than offset by the savings associated with fewer injections. By reducing vaccination regimens from three injections to one, all the associated costs such as healthcare worker time, refrigeration during transportation, needles, and syringes would decrease by two-thirds, making this a potentially viable strategy even in the developing world.

## 5 Conclusions

Here we developed pulsatile microsphere formulations that release antigen in three pulses in vitro and thereby recapitulate the kinetics of a clinical three bolus immunization schedule. We demonstrate in vivo that these microparticle formulations containing BSA as a model vaccine antigen were able to induce peak antibody titers that were non-inferior to three bolus injections administered at 0, 4, and 8 weeks. Future experiments will aim to further improve the immunogenicity of antigen-containing microspheres by using higher molecular weight PLGA to counteract accelerated in vivo degradation and approach two-month antigen release timing to more closely match the dosing regimen for many traditional vaccines. More widely-spaced bursts will allow BSA-specific plasma cell populations and associated antibody levels to subside and therefore promote an appropriate secondary immune response [30]. Future studies will also aim to encapsulate adjuvanted commercial vaccines that allow for the measurement of antigen stability and corresponding neutralizing antibody titers. Although several technical and practical challenges still remain, the results from these studies indicate that controlled release from PLGA microspheres may have the potential to streamline vaccination regimens and benefit the 19.4 million infants that remain underimmunized each year [2].

## Funding sources

This work was funded by the Bill and Melinda Gates Foundation (OPP1095790). Use of the MIT Department of Comparative Medicine's animal facility was also supported in part by the David H. Koch Institute for Integrative Cancer Research Support Grant P30-CA14051 from the National Cancer Institute.

## Conflicts of interest

The authors have no conflicts of interest to report.
